# Toward routine implementation of phage testing: an inter-laboratory comparison of a disk-based susceptibility testing (Disk-PST)

**DOI:** 10.1128/jcm.00029-26

**Published:** 2026-10-12

**Authors:** K. Ekat, L. Scharnagl, A. Hahn, S. Deinhardt-Emmer, A. Trimborn, J. Landtag, M. Wietzke, J. Elias, F. Lestin-Bernstein, W. Splettstoesser, I. Ramming, J. K. Knobloch, J. Färber, F. Szabados, A. Podbielski, P. Warnke, R. Skusa

**Affiliations:** 1 Institute of Medical Microbiology, Virology and Hygiene, University Medicine Rostock, Rostock, Germany; 2 Institute of Medical Microbiology, Jena University Hospital 39065, Jena, Germany; 3 Institute of Medical Microbiology and Hygiene, Medical Faculty of Mannheim, Heidelberg University, Mannheim, Germany; 4 MVZ Medizinische Labore Dessau Kassel GmbH, Abteilung Mikrobiologie und Molekularbiologie, Dessau, Germany; 5 State Office for Health and Social Affairs 560675 , Rostock, Germany; 6 MDI Limbach Berlin GmbH 702989 , Berlin, Germany; 7 HMU Health and Medical University 582078, Potsdam, Germany; 8 Department for Clinical Hygiene and Infectiology/Institute of Laboratory Medicine, Helios Clinics of Schwerin, University Campus of MSH Medical School Hamburg—University of Applied Sciences and Medical University, Schwerin, Germany; 9 MVZ wagnerstibbe for Medical Microbiology, Infectiology, Hygiene and Tropical Medicine, Göttingen, Germany; 10 Institute for Hygiene and Microbiology, University of Würzburg, Würzburg, Germany; 11 Institute of Medical Microbiology, Virology and Hygiene, Department for Infection Prevention and Control, University Medical Center Hamburg-Eppendorf 538358, Hamburg, Germany; 12 Institute of Medical Microbiology and Hospital Hygiene, Otto von Guericke University Magdeburg 647319 , Magdeburg, Germany; 13 Medical Laboratory Practice Osnabrück, Georgsmarienhütte, Germany; 14 European Society of Clinical Microbiology and Infectious Diseases (ESCMID) Study Group for Non-traditional Antibacterial Therapy (ESGNTA) 454626, Basel, Switzerland; Universitat Munster, Münster, Germany

**Keywords:** phage susceptibility testing, spot test, standardized phage testing

## Abstract

In the context of an emerging post-antibiotic era, phage therapy is gaining increasing attention. Standardized approaches for phage susceptibility testing (PST) are crucial to enable objective and effective phage selection for personalized treatment, yet no standardized reference method currently exists. To overcome this impediment, a standardized disk-based PST protocol was validated in-house (intra-laboratory comparison) involving 28 participants, followed by an inter-laboratory comparison across 12 laboratories in Germany with 39 participants. Preselection testing revealed high concordance between Disk-PST and Spot test, supporting the selection of strains with consistent phage susceptibility profiles for the interlaboratory comparison. An *Escherichia coli* phage, a *Staphylococcus epidermidis* phage, and a negative control (SM buffer) were tested on four bacterial strains (two *E. coli* and two *S*. *epidermidis* strains). Testing was blinded and included two technical and two biological replicates per condition. The plaque assay was performed at the coordinating laboratory (University Medical Center Rostock) and served as the reference standard. The in-house validation showed high reliability and strong agreement with the plaque assay, requiring only minor methodological adjustments prior to the inter-laboratory trial. The inter-laboratory comparison confirmed the robustness of the method, showing almost perfect agreement with the plaque assay across all participating laboratories. In the rare event of false-positive or inconclusive results, phage cross-contamination, misinterpretation of microcolonies within lysis zones, and limited information on negative controls due to the blinded test setting were causative. In accordance with its high inter-laboratory reliability, the PST-disk method seems to be highly suitable for easy implementation in routine laboratory settings. Further investigations are necessary to correlate phage test results with clinical therapy outcomes—an issue that should be addressed in future clinical trials.

**IMPORTANCE:**

The rapid global spread of multidrug-resistant bacteria has intensified the need for effective alternative therapeutic strategies, including bacteriophage therapy. Because phage activity is highly strain-specific, accurate and standardized phage susceptibility testing (PST) is essential for safe and effective clinical implementation, yet standardized protocols are currently lacking. In this nationwide, blinded inter-laboratory study, we evaluated a routine-diagnostic–adapted single-disk PST method and demonstrated its excellent agreement with the gold-standard double agar layer plaque assay. Importantly, this approach represents a significant improvement over existing PST methods by providing a standardized, reproducible, and easy-to-implement assay with high potential for widespread application in clinical routine microbiology laboratories.

## INTRODUCTION

Bacteriophages are increasingly attracting attention as a promising solution to the therapeutic dilemma posed by multidrug-resistant bacteria and complex infections, such as foreign material-associated infections. While studies in Eastern Europe indicate the efficacy of phage therapy (1, 2), Western countries lack controlled, randomized clinical trials demonstrating the effectiveness of this treatment (3, 4). The growing demand for phage therapy medicinal products (PTMPs) suitable for all potential users and applications has highlighted the lack of standardized efficacy testing against patient-specific clinical isolates so far. Antibiotics have a defined spectrum of activity and are nowadays generally easily available in acute infections by empiric antibiotic regimens, well established due to extensive historical data; nevertheless, they require susceptibility testing for targeted therapy and, in particular, in cases of clinical non-response. However, phages are more specific to their targeted bacteria than antibiotics and therefore, at present, prior laboratory testing is mandatory. This “personalized” approach is time-consuming, as it involves preparing a personalized phage cocktail containing phages shown to be active against the patient’s strain, but this approach may provide more effective phage therapy options (5, 6). Recently, a case series of 100 consecutive patients in Belgium reported an eradication rate of 61.3% using personalized phage therapy (7). Although not yet established, a comparable approach for PTMPs in empiric broad-spectrum therapy may be achievable. This would require the development of standardized and easily implementable phage susceptibility testing (PST) workflows, in combination with broad-spectrum phage cocktails. Together, these advances would represent an important step toward the broader clinical applicability of phage therapy.

Testing methods for selecting appropriate phages for personalized therapy include the most widely used agar-based methods, such as the spot test and the double agar overlay assay (8). The spot test offers decisive advantages: it allows multiple bacteriophages to be tested against a single bacterial strain simultaneously and is easy to handle. This makes it particularly useful as a rapid screening method for identifying potential therapeutic phages. However, false-positive results can occur due to a phenomenon known as “lysis from without”, which describes bacterial lysis induced by phage adsorption without successful replication. For this reason, the spot test is considered a qualitative method that requires confirmation through the double agar overlay assay (9).

In routine clinical microbiology, the primary and clinically relevant consideration is often not the efficiency of phage replication itself, but rather whether a phage consistently produces a reproducible lysis zone on a given bacterial isolate under standardized conditions. Consequently, routine phage susceptibility testing must first be reduced to an attributive readout: visible lysis versus no visible lysis. Additional assays, such as plaque assays, growth kinetics, checkerboard, and survival assays, represent the next step toward the optimized selection and characterization of phages (10).

In research and specialized laboratory settings, a positive spot test is commonly followed by a double agar overlay plaque assay. By detecting individual plaques, this assay confirms that the phage can complete the full lytic cycle (11). Furthermore, this method enables the assessment of phage activity by determining the efficiency of plating (EOP), which expresses the phage’s activity in comparison to its activity against a reference host strain. EOP has also been used to establish a classification system for phage activity against the test strain: in comparison to the lytic activity on the reference host strain, defined as 1.0, the classes high (≥0.5), moderate (0.1 ≤ EOP < 0.5), low (0.001 ≤ EOP < 0.1), and inefficient (<0.001) are discriminated (12). While the double agar overlay assay combined with EOP determination represents the quantitative reference method to assess phage replication efficiency under laboratory conditions, this level of analysis exceeds the requirements of routine diagnostic laboratories. Furthermore, both assays are not yet standardized, especially not across different target bacterial species, such as genus-related spot test. This term refers to the concept of applying a largely uniform testing setup across different bacterial genera as host strains, ideally independent of characteristics such as Gram status. Such a standardized, cross-genus approach would be analogous to agar diffusion methods used in antimicrobial susceptibility testing (AST), facilitating comparability and routine implementation.

To date, no rapid, standardized test is implemented in a “routine” clinical microbiology laboratory to assess the susceptibility of a bacterial strain towards phages (11). As a consequence, the methodology of the spot test is often altered by variations in agar medium, spot size, incubation time, and the concentrations of bacterial and phage inocula (12–15), reducing the comparability of the results between different laboratories.

In contrast, AST for antibiotics, such as the single-disk test, is fully standardized by organizations like the European Committee on Antimicrobial Susceptibility Testing (EUCAST) and the Clinical and Laboratory Standards Institute (CLSI). Currently, EUCAST breakpoints and methodologies are implemented in clinical microbiology laboratories across Europe and increasingly in other regions worldwide (16). Both the CLSI and EUCAST systems are recommended by the World Health Organization’s Global Antimicrobial Resistance Surveillance System (GLASS) (17).

A similarly standardized and harmonized testing framework would also be desirable for the investigation of phage specificity, as such data can likewise underpin crucial therapeutic decisions. The first standardized phage susceptibility test was published in 2022 (18). It is agar-based and offers the significant advantage of being easily implementable in all routine laboratories already performing antimicrobial susceptibility testing according to the EUCAST standardized disk diffusion method. In contrast to the conventional spot test, the use of cellulose disks provides a predefined and standardized application format for phages that is already familiar to routine laboratory personnel. This simplified workflow facilitates handling, reduces operator-dependent variability, and enables straightforward integration of phage susceptibility testing into existing AST infrastructures. Recently, a EUCAST subcommittee for phage susceptibility testing was established as an initiative to standardize bacteriophage testing methods and criteria, aiming to advance the integration of phage therapy into strategies addressing antimicrobial resistance (AMR) (19).

This study aims to evaluate and compare the results of a standardized phage susceptibility testing within the framework of a nationwide inter-laboratory trial involving multiple diagnostic microbiological laboratories in Germany. The objective is to assess the reproducibility, reliability, and diagnostic applicability of the method within different laboratory settings.

## MATERIALS AND METHODS

### Media, chemicals, and devices

Materials utilized for the procedures described below are provided in Table S1.

**TABLE 1:** Participating German laboratories of inter-laboratory comparison of Disk-PST

Laboratory	Location	Laboratory setting	No. of participants	
			Academics	Technicians
Department of Microbiology, MDI Limbach; Medical Laboratory Berlin	Berlin	Private medical laboratory	0	2
Department of Microbiology; Medical Laboratory Dessau	Dessau	Private medical laboratory	2	0
Department of Microbiology and Hygiene, MVZ wagnerstibbe; Medical Laboratory Göttingen	Göttingen	Private medical laboratory	1	2
Institute for Medical Microbiology, Virology and Hygiene, Department for Infection Prevention and Control; University Medical Center Hamburg-Eppendorf	Hamburg	University hospital laboratory	3	1
Group Practice for Laboratory Medicine and Microbiology; Department of Microbiology, Hameln	Hameln	Private medical laboratory	1	2
Institute of Medical Microbiology; University Hospital Jena	Jena	University hospital laboratory	2	2
Institute for Medical Microbiology and Hospital Hygiene; University Hospital Magdeburg	Magdeburg	University hospital laboratory	1	2
Institute for Medical Microbiology and Hygiene; University Hospital Mannheim	Mannheim	University hospital laboratory	2	2
Medical Laboratory Practice Osnabrück, Georgsmarienhütte	Osnabrück	Private medical laboratory	3	2
Testing Laboratory of the State Office for Health and Social Affairs Mecklenburg-Vorpommern, Rostock	Rostock	Government testing laboratory	0	3
Department of Medical Microbiology, Institute for Laboratory Medicine; Helios Clinics of Schwerin	Schwerin	Tertiary care hospital laboratory	2	2
Institute for Hygiene and Microbiology; University of Würzburg	Würzburg	University hospital laboratory	1	1

### Study design

**Fig 1:**
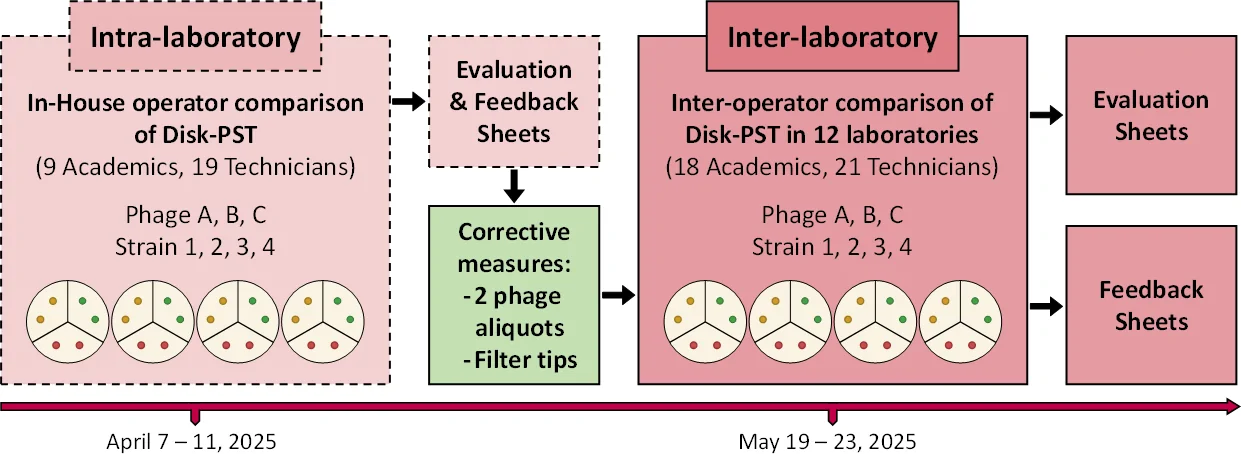
Schematic study design of the intra- and inter-laboratory comparison. In the initial intra-laboratory operator comparison (9 academics, 19 technicians), phages A, B, and C were tested against strains 1–4 using the Disk-PST. The resulting data were evaluated using evaluation and feedback sheets to determine corrective measures (including the addition of two phage aliquots per participant and the recommendation of filter tip use). In the subsequent inter-laboratory comparison, the revised protocol was evaluated in 12 participating laboratories (18 academics and 21 technicians) again using phages A, B, and C with strains 1–4. Performance outputs were collected via evaluation and feedback sheets.

Our study design is summarized in Fig. 1. Initially, the Disk-PST was evaluated in an intra-laboratory comparison in the routine laboratory of the Institute for Medical Microbiology, Virology, and Hygiene at the University Medical Center Rostock, Rostock, Germany. A total of 28 participants took part in this study, including 9 academics and 19 technicians. In this study, “academics” refers to physicians and scientists with academic training working in routine diagnostic microbiology laboratories, while “technicians” refers to technical laboratory personnel (e.g., medical laboratory assistants or biomedical laboratory technicians) performing routine diagnostic work. The Disk-PST was performed by all participants within a time span of five consecutive days. All participants received precise instructions for the Disk-PST's performance and evaluation as exemplified below. Afterward, the results of the intra-laboratory comparison were analyzed, and corrective measures were applied for the inter-laboratory comparison. Two key measures were implemented to prevent contamination: (i) each participant received two separate phage suspensions, one for each day of testing—to prevent carryover contamination between biological replicates; (ii) all laboratories were instructed to use filter pipette tips, as filter tips have been described as effective in protecting the micropipettes from sample contamination by preventing the entry of aerosols or excess liquids (20). Importantly, strain selection, phage propagation, and assay validation were performed in a stepwise manner prior to the inter-laboratory study, ensuring that the ring trial was conducted using pre-characterized phage–host combinations with defined lytic phenotypes.

For the inter-laboratory comparison, 12 routine diagnostic laboratories across Germany participated (Table 1).

A total of 39 volunteers performed the Disk-PST from 19 to 23 May 2025, including 18 academics and 21 technicians. Each laboratory received a Disk-PST test kit, containing phage aliquots for each participant, as well as all test strains. Furthermore, sterile Drigalski spatulas, sterile disks, and sterile tweezers were included in each test kit. Additional materials and equipment required were provided by the participating laboratories. All participating laboratories had access to a shared file containing a video instruction, a standard operating procedure, evaluation sheets, and feedback sheets (see Video S1 and Fig. S3 through S6). Following the inter-laboratory test, the evaluation forms and feedback forms were returned by the laboratories and statistically evaluated.

Bacteriophages, host strains, and test strains used for Disk-PST are summarized in Table 2.

**TABLE 2:** Phages and bacteria used in this study

Designation	Description
Bacteriophages	
Phage A	SM buffer (negative control)
Phage B	*Staphylococcus* phage UMR_Minerva (DSM 117679)
Phage C	*Escherichia* phage UMR_Zora (DSM 120226)
Bacteria	
Strain 1	Host strain: *Staphylococcus epidermidis* (DSM 117681)
Strain 2	Test strain: *Escherichia coli* (clinical isolate)
Strain 3	Host strain: *Escherichia coli* (DSM 120227)
Strain 4	Test strain: *Staphylococcus epidermidis* (clinical isolate)

### Bacteriophage propagation

For *Escherichia* phage and *Staphylococcus* phage enrichment, lysogeny broth (LB)-based and caso bouillon (CB)-based components (media and agar) were used, and the double agar overlay technique was utilized (21). The bacterial host strains (*E. coli* DSM 120227 or *S. epidermidis* DSM 117681) used for phage propagation corresponded to strains 1 and 3 included in the inter-laboratory comparison to ensure robust and reproducible phage production and a clearly lytic positive control within the study design.

After incubation of the agar plates with a lawn of host bacteria and the appropriate phages at 37°C for 18 h under ambient conditions, 4 mL of SM buffer was added to each plate and gently spread across the surface using a rotating platform for 5 h. The supernatant was then centrifuged (2,772 × *g*, 5 min, 15°C) and double sterile-filtered (0.45 and 0.22 μm). The enriched phage suspension was stored at 6°C. Phage concentration was adjusted by serial dilution in SM buffer to 10^8^ plaque-forming units per mL (PFU/mL) for *Staphylococcus* phage B and 10^11^ PFU/mL for *Escherichia* phage C.

### Phage stability assessment

The stability of phages was evaluated by incubating phage suspensions under various storage conditions and assessing the remaining infectivity over time. Phages with an initial concentration of 10^9^ PFU/mL were diluted 1:10 in different storage media, resulting in a final concentration of 10^8^ PFU/mL. The media employed for dilution included SM buffer, SM buffer enriched with glycerol (1:10), 0.9% NaCl, and 0.9% NaCl enriched with glycerol (1:10).

The prepared phage suspensions were aliquoted and stored at four different temperatures: 21–24°C, 4 °C, −20°C, and −80°C. Samples were collected from each condition after 1, 3, 7, and 14 days of storage to evaluate the remaining phage concentration using the double agar overlay technique.

### Strain selection

### Spot test

Prior to the intra- and inter-laboratory comparison, overall phage susceptibility testing of the bacterial strains was carried out on 51 *S. epidermidis* and 132 *E. coli* clinical isolates (see Fig. S1 and S2). Therefore, bacteria were cultivated overnight in the corresponding medium at 37°C, 5% CO_2_. Soft agar was mixed with 100 μL of the reference bacterial suspension and promptly poured onto bottom agar plates. Once the top agar containing the bacteria solidified, 10 μL of the phage suspension (≥10^6^ PFU/mL) was applied to the surface and allowed to dry. The plates were then incubated for 18 h at 37°C under ambient conditions and subsequently examined for lysis zones. The spot test method was performed, and lysis zones were evaluated according to reference 13.

### Double agar overlay technique

The double agar overlay assay was performed using 100 μL of bacterial culture (OD_600_ = 0.5–0.8) and 100 μL of the corresponding phage concentrate/dilution. A series of four dilutions is typically tested, usually extending up to a dilution factor of 10^−9^. Both the bacterium and phage were added to 4 mL of liquid soft agar. The mixture was vortexed and immediately poured onto bottom agar plates. After solidification of the top agar at 21–24°C for 5 min, plates were incubated for 18 h at 37°C with a 5% CO_2_ atmosphere (21).

This assay was conducted at the University Medical Center Rostock in a specialized laboratory setting as a reference method to confirm productive phage replication via plaque formation during the initial strain screening. In this study, it was used to support strain selection and characterization and for phage propagation. No double agar overlay assays were performed by routine diagnostics laboratories.

### Efficiency of plating

EOP was determined by quantifying PFU using the double agar overlay technique. The phage concentration in PFU/mL was assessed through serial dilution. PFU values obtained from phage/test strain combinations were then expressed relative to the phage concentration determined on the original host strain (12, 13).

Importantly, efficiency of plating (EOP) determinations were performed exclusively during the initial in-house screening phase to support the selection of representative phage–host combinations. This preselection step was used to identify bacterial strains with defined lytic phenotypes for subsequent reproducibility testing. No EOP analyses were performed during either the in-house or inter-laboratory comparison, as the study was intentionally designed to focus on attributive outcomes (lysis vs no lysis) under standardized testing conditions.

### Disk-PST under routine diagnostic conditions

Disk-PST was performed under standardized routine laboratory conditions, following a published protocol by Skusa et al. (18). The standardized direct colony suspension method was used for inoculum preparation. Therefore, the bacterial strain to be tested was cultured overnight on universal growth medium (Columbia agar) at 35 ± 1°C. A single colony was harvested using a sterile inoculation loop.

Subsequently, the harvested colony was suspended in saline solution (0.9%) and mixed thoroughly to achieve uniform turbidity. The bacterial suspension was then adjusted to a turbidity equivalent to the 0.5 McFarland standard (acceptable range: 0.45–0.55) following the EUCAST disk diffusion method by either diluting with saline or adding additional bacterial material (22).

Prior to inoculation, Mueller-Hinton (MH) agar plates were equilibrated to 21–24°C. To ensure uniform bacterial lawn formation, a 100 µL aliquot of the adjusted bacterial suspension was evenly spread onto the surface of each plate using a Drigalski spatula.

Phage application was conducted using sterile cellulose disks (Ø 6 mm). The disks were firmly pressed onto the inoculated agar surface using sterile forceps, ensuring full and uniform contact without repositioning after placement. A maximum of eight disks was applied per plate to prevent overlapping lysis zones. Subsequently, 50 µL of the phage suspension under investigation was carefully pipetted onto each disk, using sterile filtered tips and avoiding direct contact with the agar surface.

After phage application, plates were allowed to dry for 5 min at 21–24°C, avoiding sudden plate movements. The plates were then incubated at 35 ± 1°C for 18–24 h under ambient atmospheric conditions. Plates were not inverted during incubation.

**Fig 2:**
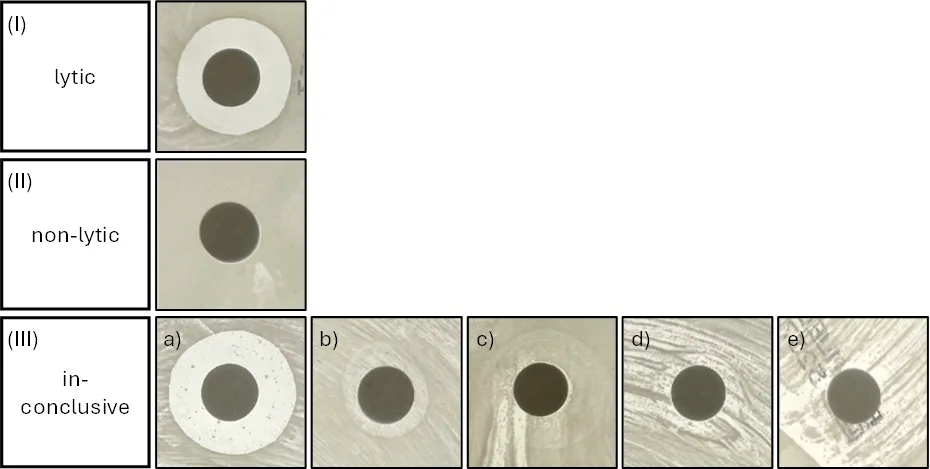
Representative Disk-PST readouts and interpretation criteria. Results were classified as (I) lytic when clear, well-defined zones of complete bacterial clearance surrounded the phage disk, as (II) non-lytic when no visible clearing was observed, and as (III) inconclusive when ambiguous patterns occurred: (a) microcolonies within the cleared area, (b) faint or (c) irregular lysis zones, or (d) thin and (e) non-uniform bacterial lawn. Images were captured using a smartphone camera on an LED light box under standardized lighting conditions. Images were cropped and uniformly adjusted for brightness and contrast using Microsoft PowerPoint LTSC (Microsoft Corp., Redmond, WA, USA).

Disk-PST readouts were interpreted based on the presence and morphology of lysis zones surrounding the phage-applied disks. Results were classified as lytic when clear, well-defined complete zones of bacterial clearance were observed. In contrast, non-lytic outcomes were defined by the absence of visible clearing. An additional category, inconclusive, was introduced to account for ambiguous patterns (e.g., faint, irregular, or presence of microcolonies) and to capture variability arising from limited operator experience in phage testing. Representative examples of each category are provided in Fig. 2. A stepwise workflow protocol for Disk-PST under routine conditions is provided in Table 3.

**TABLE 3:** Disk-PST step-by-step workflow summarizing procedure, key parameters, and critical considerations

Step	Procedure	Key parameters	Critical considerations
1	Overnight culture of bacterial strain	Universal growth medium (e.g., Columbia agar), 35 ± 1°C	Use fresh colonies
2	Preparation of bacterial suspension	0.9% NaCl	Homogeneous mixing required
3	Turbidity adjustment	0.5 McFarland (0.45–0.55)	According to EUCAST disk diffusion method
5	Agar inoculation with bacterial strain	100 µL spread by spatula	Uniform lawn essential
6	Disk placement	Sterile cellulose disks (Ø 6 mm)	Firm contact, no repositioning
7	Phage application	50 µL per disk	Avoid agar contact
8	Drying	5 min at RT	Avoid plate movement
9	Incubation	35 ± 1°C, 18–24 h	Plates not inverted
10	Readout	Lysis zones	See interpretation criteria in Fig. 2

### Assessment of concordance between double agar overlay technique and Disk-PST

Concordance between the Disk-PST results obtained in the interlaboratory comparison and the gold-standard assay was assessed by comparing the categorical Disk-PST outcome with the corresponding plaque formation pattern in the double agar overlay assay. For this comparison, the double agar overlay assay was categorized based on the presence or absence of single plaques. Overall categorical agreement was defined as concordance between a lytic Disk-PST result and plaque formation in the double agar overlay assay, or between a non-lytic Disk-PST result and the absence of plaques. Thus, plaque formation was interpreted as a lytic result, whereas the absence of plaques was interpreted as a non-lytic result. Inconclusive Disk-PST results were considered non-concordant.

### In-house operator comparison of Disk-PST

Based on in-house phage specificity testing (spot test and PST) of 132 *Escherichia* and 51 *Staphylococcus* clinical isolates (Fig. 1; Fig. S1), one representative lytic test strain per species was selected for interlaboratory comparison. The *E. coli* test strain exhibited an EOP of 10^−6^ PFU/mL, whereas the *S. epidermidis* strain showed an EOP of 0.14. These values were used exclusively for strain selection and were not part of the inter-laboratory performance evaluation.

**Fig 3:**
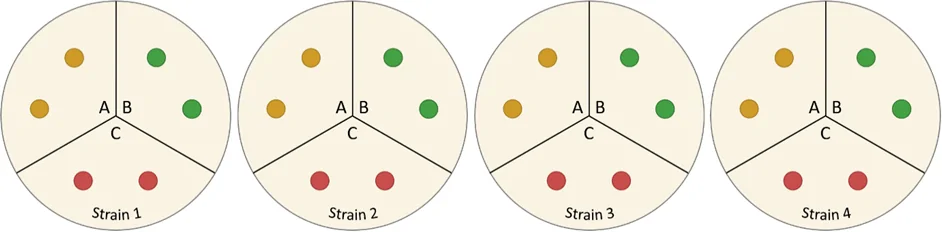
Plate layout for testing blinded phage samples (A–C) against four bacterial strains. (A) SM buffer (negative control), (B) UMR_Minerva DSM 117679, and (C) UMR_Zora DSM 120226. Strain 1, *S. epidermidis* DSM 117681; Strain 2, *E. coli* (clinical isolate); Strain 3, *E. coli* DSM 120227; Strain 4, *S. epidermidis* (clinical isolate).

Prior to the initiation of the inter-laboratory comparison, an in-house test of the Disk-PST, as described above, was conducted at the Institute of Medical Microbiology, Virology, and Hygiene, University Medical Center Rostock. Participant-specific Disk-PST kits were prepared. The phage concentrations used were 10^7^ PFU/mL for *Staphylococcus* phage B and 10^11^ PFU/mL for *Escherichia* phage C. Additionally, overnight cultures of the test strains were prepared on Columbia agar. In order to ensure that all 28 participants (19 technicians and 9 academics) were adequately prepared, they were provided with a video tutorial explaining the Disk-PST procedure. The phage-specific testing was carried out in accordance with the requirements of a single-blinded study. The allocation of phage suspensions and the layout of agar plates are illustrated in Fig. 3.

For each test strain, phage suspensions A, B, and C were tested in duplicate (technical replicates). Each participant repeated the Disk-PST on a second experimental day (biological replicate), using the same participant-specific phage aliquots on both days. Test evaluation was conducted on the following day by each investigator using a standardized evaluation sheet (see Fig. S5). In addition, each participant completed a pre-structured feedback form to assess the test procedure (data not shown).

### Inter-laboratory operator comparison of Disk-PST

Participating laboratories are listed in Table 1. All participating laboratories have experience in standardized susceptibility testing according to EUCAST guidelines as part of routine diagnostic activities in clinical microbiology for patient care (22). Phage propagation was carried out as previously described during the period from 12 to 14 May 2025. Subsequent quantification of the generated phage suspensions was performed using the double agar overlay technique. Final concentrations were determined to be 10^9^ PFU/mL for *Staphylococcus* phage B and 10^11^ PFU/mL for *Escherichia* phage C.

Bacterial test strains were prepared as overnight cultures on Columbia blood agar at 35 ± 1°C for 18–24 h under CO_2_-enriched aerobic conditions. Colony material was collected using a flock swab (Σ-TRANSWAB, Medical Wire) and packaged accordingly. Sample shipment was conducted at ambient temperature via a certified logistics provider on 16 May 2025. Delivery to all participating laboratories was confirmed by 19 May 2025. The inter-laboratory comparison was carried out until 23 May 2025.

### Statistical analysis

All statistical analyses were performed using Stata BE 19.5. The evaluation of diagnostic performance was conducted using descriptive statistics. In order to compare proportions of disagreement across different testing days (biological replicate) and between duplicates (technical replicate), tests of proportions were applied. Contingency tables assessed with Fisher’s exact test were further examined. Moreover, the interrater agreement of the test results was calculated using Fleiss' Kappa (*κ*), in comparison to the gold standard (double agar overlay assay). The double agar overlay assay was only performed at University Medical Center Rostock. The following classification is employed to measure the strength of agreement: 0–0.01 poor agreement, 0.01–0.20 slight agreement, 0.21–0.40 fair agreement, 0.41–0.60 moderate agreement, 0.61–0.80 substantial agreement, and 0.81–1.00 almost perfect agreement (23). To explore potential factors influencing deviations, subgroup analyses were performed stratified by the role of the testing personnel, the strain, and phage type. Associations were assessed using Fisher’s exact test, considering two-sided *P* values. A significance level of *P* = 0.05 was defined for all calculations, but *P* values were not adjusted for multiple testing.

## RESULTS

### Phage stability characterization

The stability of the phage preparations was assessed under four storage temperatures (21–24°C, 4°C, −20°C, and −80°C) in different media (SM buffer, SM buffer enriched with glycerol [1:10], 0.9% NaCl, and 0.9% NaCl enriched with glycerol [1:10]) over a period of 14 days (see Fig. S1). The most stable preparation conditions for both phages were identified as SM buffer and room temperature; therefore, these conditions were selected for the intra- and inter-laboratory comparison.

### Selection and characterization of strains for the interlaboratory comparison

To select suitable wild-type strains for inclusion in the interlaboratory comparison, Disk-PST and Spot tests were performed in parallel using the respective replicates for 132 *E. coli* strains and 51 *S. epidermidis* strains (Tables S2 and S3). Concordance between the two methods was assessed based on positive and negative agreement, which were 100% and 98.3%, respectively, for *E. coli* and 100% and 78.8% for *S. epidermidis*. For the selected strains, the presence of individual plaques was subsequently confirmed using the double agar overlay assay to rule out the phenomenon of lysis from without. Finally, the efficiency of plating (EOP) was determined relative to the respective reference strain to further characterize the phage–host interaction. These data were used to identify strains showing consistent results across the two phage susceptibility testing approaches for inclusion in the interlaboratory comparison.

### Assessment of Disk-PST reliability under controlled in-house conditions

The results of the in-house Disk-PST series were evaluated against the gold standard defined by double agar overlay plaque assay performed in our research laboratory. Across both test days (biological replicates 1 and 2), both technical replicates performed by 28 participants showed almost perfect agreement with the reference values (*κ* values of 0.973 and 0.849, respectively). Sensitivity and specificity were 97.32% and 99.55%, on test day 1, and 97.32% and 90.62% on test day 2, with consistently high predictive values (PPV ≥ 87.20%, NPV ≥ 99.11%). In Table 4 and Table 5, an overview of the absolute test outcomes and summary of the corresponding statistical performance parameters is provided.

**TABLE 4:** Comparison of in-house Disk-PST results with the gold standard for each test day and technical replicate^*a*^

Disk-PST	Gold standard		Total
	Non-lytic (no plaques)	Lytic (plaques present)	
BR 1/TR 1			
Non-lytic	223	2	225
Lytic	1	109	110
Inconclusive	0	1	1
Total	224	112	336
BR 1/TR 2			
Non-lytic	223	2	225
Lytic	1	109	110
Inconclusive	0	1	1
Total	224	112	336
BR 2/TR 1			
Non-lytic	203	1	204
Lytic	15	109	124
Inconclusive	6	2	8
Total	224	112	336
BR 2/TR 2			
Non-lytic	203	1	204
Lytic	16	109	125
Inconclusive	5	2	7
Total	224	112	336

^*a*^ BR, biological replicate; PST, phage susceptibility test; TR, technical replicate.

**TABLE 5:** Statistical performance parameters of Disk-PST compared to the gold standard across different test days and technical replicates^*a*^

Test result	Fleiss kappa (CI)	Sensitivity	Specificity	PPV	NPV
BR 1/TR 1	0.973 (0.947–1.000)	97.32	99.55	99.09	99.11
BR 1/TR 2	0.973 (0.943–0.993)	97.32	99.55	99.09	99.11
BR 2/TR 1	0.849 (0.785–0.888)	97.32	90.62	87.90	99.51
BR 2/TR 2	0.848 (0.798–0.904)	97.32	90.62	87.20	99.51

^*a*^ BR, biological replicate; CI, confidence interval; NPV, negative predictive value; PPV, positive predictive value; TR, technical replicate.

When comparing the biological replicates, a significantly higher error rate was observed on test day 2 (7.14 %) compared to test day 1 (1.19 %), with a *z*-score of −3.86 and *P* < 0.001. For determination of within-day test reproducibility, technical replicates were compared. On both days, identical error rates between technical replicates 1 and 2 were observed (*P* = 1.00 for both comparisons). An overview of replicate-specific error rates is provided in Table S4 (biological replicates) and Fig. S1 (technical replicates).

While these results confirm the overall robustness and reliability of the Disk-PST, the deviation rate from the gold standard varied depending on the tested bacterial strains and phages. With respect to strain-specific deviations, no discrepancies occurred for strains 1–3 on test day 1, whereas strain 4 showed a significant deviation rate of 4.76% (*P* = 0.015). On test day 2, deviations were observed across all strains, with higher rates for strains 2 and 3 (10.71% and 11.90%) compared to strain 4 (3.57%) and strain 1 (2.38%) (*P* = 0.029). Similarly, phage-dependent variability was observed. On test day 1, deviation rates were low across all phages (≤2.68%). On test day 2, phage B showed a significantly higher deviation rate (19.64%) compared to phages A and C (0.00% and 1.79%, respectively). Full results are shown in Table S6 and Table S7.

### Inter-laboratory operator variability

#### Performance of the Disk-PST compared to the gold standard

A total of 39 participants from 12 routine diagnostic laboratories evaluated the Disk-PST, and results were compared against the gold standard defined by the double agar overlay plaque assay performed in our laboratory.

Across all four datasets (two test dates, each with two technical replicates), the Disk-PST showed an excellent level of agreement with the reference method. *κ* values ranged from 0.925 to 0.939, indicating a consistently strong concordance. Sensitivity values varied between 92.25% and 94.87%, while specificity remained high throughout (97.76–98.72%). Predictive values were similarly robust with a PPV between 98.67% and 100.00%, and NPV ranging from 99.35% to 100.00%. The number of inconclusive results remained low across all replicates. Inconclusive results were predominantly characterized by the presence of microcolonies within otherwise clear lysis zones, indicating partial bacterial regrowth or heterogeneous phage susceptibility (see Fig. 2).

Absolute test outcomes are shown in Table 6, whereas statistical performance metrics are listed in Table 7.

**TABLE 6:** Comparison of inter-laboratory Disk-PST results with the gold standard for each test day and technical replicate^*a*^

Disk-PST	Gold standard		Total
	Non-lytic (no plaques)	Lytic (plaques present)	
BR 1/TR 1			
Non-lytic	307	2	309
Lytic	2	148	150
Inconclusive	3	6	9
Total	312	156	468
BR 1/TR 2			
Non-lytic	305	2	307
Lytic	1	147	148
Inconclusive	6	7	13
Total	312	156	468
BR 2/TR 1			
Non-lytic	308	0	308
Lytic	0	144	144
Inconclusive	4	12	16
Total	312	156	468
BR 2/TR 2			
Non-lytic	307	1	308
Lytic	1	145	146
Inconclusive	4	10	14
Total	312	156	468

^*a*^ BR, biological replicate; PST, phage susceptibility test; TR, technical replicate. Inconclusive results were categorized as depicted in Fig. 2 (III).

**TABLE 7:** Statistical performance parameters of inter-laboratory Disk-PST compared to the gold standard across different test days and technical replicates^*a*^

Test result	Fleiss kappa (CI)	Sensitivity (95% CI)	Specificity (95% CI)	PPV (95% CI)	NPV (95% CI)
BR 1/TR 1	0.939 (0.906–0.966)	94.87 (90.14–97.78)	98.40 (96.30–99.48)	98.67 (95.27–99.84)	99.35 (97.68–99.92)
BR 1/TR 2	0.925 (0.887–0.957)	94.23 (89.33–97.33)	97.76 (95.43–99.09)	99.32 (96.29–99.98)	99.35 (97.67–99.92)
BR 2/TR 1	0.925 (0.886–0.954)	92.31 (86.95–95.96)	98.72 (96.75–99.65)	100.00 (97.47–100)	100.00 (98.81–100)
BR 2/TR 2	0.925 (0.883–0.951)	92.95 (87.73–96.43)	98.40 (96.30–99.48)	99.32 (96.24–99.98)	99.68 (98.20–99.99)

^*a*^ BR, biological replicate; CI, confidence interval; NPV, negative predictive value; PPV, positive predictive value; TR, technical replicate.

#### Reproducibility across biological and technical replicates

When comparing the biological replicates, a higher error rate of 3.41% was observed on test day 2 compared to 2.79% on test day 1 (*z* = −0.57, *P* = 0.571). To assess within-day test reproducibility, technical replicates were compared for each test day. On day 1, error rates were 2.78% and 3.41% in replicates 1 and 2, respectively (*P* = 0.571). On day 2, both replicates showed identical error rates of 3.41% (*P* = 1.00). An overview of replicate-specific error rates, confidence intervals, and statistical comparisons is provided in Table 8 (biological replicates) and Table 9 (technical replicates).

**TABLE 8:** Comparison of error rates between biological replicates during inter-laboratory comparison^*a*^

BR	Total samples	Errors	Error rate	95% CI	*z*-Score	*P* value
1	468	13	2.78%	1.29–4.28%	−0.57	*P* = 0.571
2	468	16	3.41%	1.78–5.07%		

^*a*^ BR, biological replicate; CI, confidence interval.

**TABLE 9:** Comparison of error rates between technical replicates within each test day during inter-laboratory comparison^*a*^

BR	TR	Total samples	Errors	Error rate	95% CI	*z*-Score	*P* value
1	1	468	13	2.78%	1.29–4.28%	−0.57	*P* = 0.571
	2	468	16	3.41%	1.78–5.07%		
2	1	468	16	3.41%	1.78–5.07%	0.00	*P* = 1.00
	2	468	16	3.41%	1.78–5.07%		

^*a*^ BR, biological replicate; CI, confidence interval; TR, technical replicate.

#### Performance across bacterial strains and phages

Analysis of strain-specific performance revealed notable differences between isolates. While strains 1–3 showed consistently low deviation rates (0.85–1.71 %), strain 4 exhibited markedly higher rates across all test days and replicates (7.69–9.40%). These differences were statistically significant (Fisher’s exact test, *P* ≤ 0.006). Detailed results are summarized in Table 10.

**TABLE 10:** Deviation rates of the Disk-PST from the gold standard by test strain during inter-laboratory comparison^*a*^

Disk-PST	Strain				Total	Fisher’s exact
	1	2	3	4		
BR 1/TR 1						
No deviation	116 (99.15%)	116 (99.15%)	115 (98.29%)	108 (92.31%)	455 (97.22%)	*P* = 0.006
Deviation	1 (0.85%)	1 (0.85%)	2 (1.71%)	9 (7.69%)	13 (2.78%)	
BR 1/TR 2						
No deviation	116 (99.15%)	115 (98.29%)	115 (98.29%)	106 (90.60%)	452 (96.58%)	*P* = 0.003
Deviation	1 (0.85%)	2 (1.71%)	2 (1.71%)	11 (9.40%)	16 (3.42%)	
BR 2/TR 1						
No deviation	116 (99.15%)	115 (98.29%)	115 (98.29%)	106 (90.60%)	452 (96.58%)	*P* = 0.003
Deviation	1 (0.85%)	2 (1.71%)	2 (1.71%)	11 (9.40%)	16 (3.42%)	
BR 2/TR 2						
No deviation	116 (99.15%)	115 (98.29%)	115 (98.29%)	106 (90.60%)	452 (96.58%)	*P* = 0.003
Deviation	1 (0.85%)	2 (1.71%)	2 (1.71%)	11 (9.40%)	16 (3.42%)	

^*a*^ Deviation rates of the Disk-PST from the gold standard by tested strain during inter-laboratory comparison are displayed across two test days and replicates. Values are given as absolute numbers and percentages. Statistical significance was assessed using Fisher’s exact test. BR, biological Replicate; PST, phage susceptibility test; TR, technical replicate.

Following the strain-specific analysis, phage-dependent performance was assessed. In the inter-laboratory comparison, deviation rates of the Disk-PST from the gold standard were low across all phages and replicates (1.92–5.13%). No statistically significant differences were observed between phages on any test day (*P* ≥ 0.332). Detailed results are presented in Table 11.

**TABLE 11:** Deviation rates of the Disk-PST from the gold standard by phage during inter-laboratory comparison^*a*^

Disk-PST	Phage			Total	Fisher’s exact
	A	B	C		
BR 1/TR 1					
No deviation	153 (98.08%)	151 (96.79%)	151 (96.79%)	455 (97.22%)	*P* = 0.826
Deviation	3 (1.92%)	5 (3.21%)	5 (3.21%)	13 (2.78%)	
BR 1/TR 2					
No deviation	153 (98.08%)	151 (96.79%)	148 (94.87%)	452 (96.58%)	*P* = 0.332
Deviation	3 (1.92%)	5 (3.21%)	8 (5.13%)	16 (3.42%)	
BR 2/TR 1					
No deviation	153 (98.08%)	148 (94.87%)	151 (96.79%)	452 (96.58%)	*P* = 0.332
Deviation	3 (1.92%)	8 (5.13%)	5 (3.21%)	16 (3.42%)	
BR 2/TR 2					
No deviation	153 (98.08%)	149 (95.51%)	150 (96.15%)	452 (96.58%)	*P* = 0.530
Deviation	3 (1.92%)	7 (4.49%)	6 (3.85%)	16 (3.42%)	

^*a*^ Deviation rates of the Disk-PST from the gold standard by phage during inter-laboratory comparison are displayed across two test days and replicates. values are given as absolute numbers and percentages. statistical significance was assessed using Fisher’s exact test. BR, biological replicate; PST, phage susceptibility test; TR, technical replicate.

#### Feedback sheets

After the interlaboratory test had been carried out in all laboratories, each participant submitted feedback on the Disk-PST using a questionnaire. Detailed data on this can be found in Table S8 in the supplemental material. In summary, it was determined that 61.5% of participants were not familiar with phage susceptibility testing in general. In general, 97.4% are confident using the AST method. All participants found the Disk-PST easy to perform using the accompanying standard operating procedure. The results were clearly interpretable for 92.3% of the participants.

## DISCUSSION

Unlike conventional antibiotic susceptibility testing, there is a lack of standardized methodologies or validated interpretation criteria for assessing the susceptibility of bacterial strains to phages (11). Given the typically narrow host range of most phages, testing of the patient-specific pathogen responsible for the infection is essential before initiating personalized phage therapy (24). Consequently, establishing a standardized, rapid, and clinically applicable PST protocol is critical for the safe and effective integration of phage-based treatments into clinical practice (25).

The present inter-laboratory study of the standardized Disk-PST (18) demonstrated excellent agreement with the reference method (double agar overlay assay) across 12 independent routine diagnostic laboratories with an overall of 39 participants. Therefore, the study results confirm high sensitivity, reproducibility, and predictive values. Consistently low values of inconclusive results underscore the robustness and reproducibility of the Disk-PST, demonstrating its potential as a reliable test for routine phage susceptibility testing. Feedback analysis showed that all participants of the inter-laboratory study agreed that the Disk-PST is easy to perform. Only 7.7% encountered difficulties interpreting the results. We are aware that the inclusion and proportion of negative controls, particularly cross-species combinations, influence the distribution of test outcomes and thereby affect prevalence-dependent performance metrics such as predictive values. However, these controls were intentionally incorporated to enable a comprehensive assessment of assay specificity, which represents a key strength of the study design. While this approach may not fully reflect real-world prevalence, future studies incorporating a broader range of within-species phage–host combinations and applying prevalence-adjusted analyses could further complement our findings and enhance their generalizability.

Prior to the inter-laboratory comparison, the Disk-PST underwent in-house validation, displaying high reliability and strong agreement with the reference method, as well as consistently high sensitivity and specificity, particularly on the first test day. Although performance declined slightly on the second test day, overall diagnostic accuracy remained high. While technical replicates were consistent, biological replicates exhibited increased error rates on the second test day, mainly attributable to phage B (UMR_Minerva DSM 117679) and strain 4 (*S. epidermidis* clinical isolate). Based on these findings, the test setup was adapted before the inter-laboratory comparison (Fig. 1).

To date, inter-laboratory comparisons of phage susceptibility testing have been scarce. Parmar et al. conducted a comparative study involving three laboratories to assess *Pseudomonas aeruginosa* phage susceptibility testing (26), while a subsequent study by the same group (27) evaluated two different liquid-based high-throughput methods. In another study, Cunningham et al. investigated phage susceptibility of *Escherichia coli* and *Staphylococcus aureus* phages using a liquid assay approach across two laboratories, with data analysis performed via specialized software tools (28). To the best of our knowledge, the present study represents the first inter-laboratory comparison involving a dozen external laboratories and, furthermore, one of the first to employ an agar-based Disk-PST approach.

Comparable interlaboratory evaluations have been conducted in the field of antibiotic susceptibility testing (AST). Deckers et al. (29) evaluated the performance of temocillin susceptibility testing, including disk diffusion and ETEST, in 12 Belgian laboratories using broth microdilution as the reference method (29). Their multicenter study achieved excellent essential agreement rates (91.5–100%). Highest categorical agreement was achieved by ETEST (97.5%) and disk diffusion (91.6%). These metrics are analogous to those used in our study and underscore the robustness of the disk PST, which demonstrated similarly high reproducibility and diagnostic accuracy. Deckers et al. (29) emphasized that standardization of the AST improves comparability between laboratories and ensures diagnostic reliability. Their harmonized multicenter approach—using identical bacterial panels, materials, and EUCAST interpretation criteria—resulted in high reproducibility rates and categorical agreement rates (29). These considerations motivated the introduction of our proposed standardized Disk-PST (18), with the aim of achieving a similar level of reproducibility and reliability in testing phage susceptibility. To ensure robustness and reproducibility, each experiment included two technical and two biological replicates, as recommended by Pollard et al. (30). Technical replicates accounted for handling variability, while biological replicates captured variability between independent preparations of the same strain. This replication strategy strengthens the assay’s suitability for clinical microbiology laboratories (30).

During the intra-laboratory trial, we encountered sources of false-positive or inconclusive results: (i) cross-contamination between phage suspensions and (ii) interpretation challenges due to difficulties assessing microcolonies within lysis zones. Contamination represents a particular concern in phage-based assays due to the self-amplifying nature of phages, where even low numbers of infectious particles can lead to substantial propagation in contrast to AST. This underscores the need for stringent quality control and standardized workflows.

To address the problem of cross-contamination of phage suspensions, we considered adjusting the input concentrations to equal levels. However, this led to poorly defined lysis zones when testing the *E. coli* phage on both the reference and test strains, characterized by the presence of microcolonies or regrowth of a thin bacterial lawn during incubation. Variations in bacterial growth kinetics and cell size result in distinct lawn morphologies, which can significantly impact the outcome of phage susceptibility testing. Despite identical inoculation (0.5 McFarland standard) and incubation conditions, notable differences in lawn density, particularly between gram-negative bacteria (e.g., *Escherichia coli*) and gram-positive bacteria (e.g., *Staphylococcus epidermidis*), can be detected. These differences necessitate adjustments in phage concentrations to reliably produce macroscopically visible lysis zones—an issue we addressed in our inter-laboratory comparison setup.

Alternatively, inoculation protocols could be adapted based on Gram-stain characteristics. A precedent for this approach can be found in the EUCAST disk diffusion methodology (22). While the initial bacterial suspension is standardized to 0.5 McFarland for both gram-positive and gram-negative bacteria, the transfer step using a sterile cotton swab is tailored toward gram-positive and gram-negative growth behavior, respectively. This is underscored by the inoculum effect, as it may lead to an overestimation of MIC values for antibiotic susceptibility testing from a clinical point of view (31). The inoculum effect appears to be also relevant in agar-based phage susceptibility testing, where it may lead to underestimation of phage susceptibility and remains a challenge for standardization across different host strains. For our standardized experimental setup, a uniform agar inoculation protocol was required, without host-specific adjustments to inoculum density. Therefore, we pivoted from our previously proposed inoculation density of McFarland standard 0.2–0.5 (18). To compensate for the varying lawn densities, we furthermore modified the phage volume per disk to 50 µL and phage concentrations accordingly. In addition, all participating laboratories of the inter-laboratory trial received two separate phage-suspensions per participant and were advised to use filter pipette tips. Since this error did not occur during the inter-laboratory comparison, the implemented measures were considered effective in preventing this type of contamination.

Although these measures effectively reduced cross-contamination as a cause of unclear results, uncertainty remained in the interpretation of lysis zones where microcolonies had developed. One advantage of agar-based phage testing is the ability to detect phage-resistant microcolonies within lysis zones or at the edges of plaques. The regrowth of bacteria in previously clear zones consistent with initial phage sensitivity may indicate either pre-existing resistance or the emergence of resistance mechanisms *in vitro* (11). However, confirmation of phage-resistant mutants requires additional testing (32). In routine diagnostics, where AST predominates, the occurrence of microcolonies is often interpreted as an indicator of resistance mechanisms (33). Even when MIC values fall within the susceptible breakpoint range, the presence of such growth should prompt confirmatory testing in AST (34).

Due to the potential overlap in interpretation criteria between antibiotic and phage susceptibility testing, lysis zones with accompanying microcolonies were considered inconclusive by some participants in our study. Targeted training sessions and the provision of visual reference materials depicting different types of lysis patterns may help improve consistency in interpretation, particularly given that many participants had no prior experience with Disk-PST.

The fact that our agar-based Disk-PST method yields results comparable to the spot assay carries the risk of the “lysis from without” phenomenon. Consequently, the method cannot confirm the occurrence of a full phage replication–infection–lysis cycle (11). In our study, we tested each phage not only against its corresponding host strain but also against an additional test strain with clearly different EOP values (*E. coli* test strain EOP = 10^−6^; *S. epidermidis* test strain EOP = 0.14).

The role of EOP in therapeutic studies is still poorly understood. Phages with low EOP are often excluded from therapy in advance, as a minimum EOP is considered therapeutically acceptable (7, 35). Whether a specific EOP value truly influences clinical response remains to be clarified in controlled therapeutic trials (36). In the context of this study, EOP determination was not part of the Disk-PST evaluation but was used exclusively during internal strain selection to identify strains with clearly distinguishable lysis behavior. Parameters such as reference strain choice and EOP determination therefore belong to specialized phage characterization and are intentionally separated from the routine PST workflow evaluated in this inter-laboratory study.

The Israeli phage concept emphasizes the complex selection of suitable phages as critical for enabling treatment, beginning with plaque assays and incorporating additional methods such as liquid kinetic assays and checkerboard tests in subsequent steps. A key advantage highlighted in their protocol is the use of the spot assay for initial screening of large phage collections, offering a relatively fast and low-effort method (10). A similar approach was considered by Glonti and Pirnay, in which spot test methods are applied as a preliminary qualitative phage test preceding the double agar overlay plaque assay for quantitative confirmation of bacterial lysis (9). In a case study by Ferry et al. (37), a “phagogram” was performed prior to phage treatment of three patients with relapsing *Staphylococcus aureus* prosthetic knee infection utilizing spot test and liquid kinetic assay to evaluate lytic activity of the administered PTMPs (37).

We also recognize the advantages of the spot assay. Through the proposed adaptation of the spot assay method, additional resources could be mobilized for broad phage testing by routine diagnostic laboratories, which already implement standardized protocols such as those from EUCAST. Although treatment decisions should not be based solely on the results of this phage susceptibility test without confirmatory assays, they provide a valuable initial overview of potentially applicable phages.

Although the classical spot test remains a valuable screening method, its execution varies considerably between laboratories with respect to phage application and handling. The Disk-PST therefore does not aim to replace the biological principle of the spot assay but to provide a simplified and standardized workflow that can be readily integrated into routine diagnostic laboratories. The use of cellulose disks introduces a familiar handling concept analogous to established disk diffusion workflows, thereby reducing operator-dependent variability and facilitating implementation and training. While this modification may appear technically simple, simplification of laboratory procedures is a key prerequisite for successful routine implementation. In addition, the disk-based format creates opportunities for future ready-to-use phage-loaded disks and combined phage-antibiotic susceptibility testing.

A limitation of our study is that the inter-laboratory comparison was restricted to a small number of phage-strain combinations, which does not fully represent the natural diversity of phage susceptibility observed among clinical isolates. Although we complemented this by including a broader panel of isolates by in-house analyses, the simplified design of the ring trial may not capture the full variability encountered in routine clinical settings. Nevertheless, this reduction was necessary to ensure feasibility and standardization across participating laboratories.

While the Disk-PST is conceptually inspired by the standardized workflow of disk diffusion antibiotic susceptibility testing, this comparison is intended purely as an operational analogy for routine laboratory implementation and not as a methodological equivalence. The spot test is a well-established and widely used method in bacteriophage testing. Our proposed Disk-PST builds on this principle and also includes the step of phage spotting onto the disk surface instead of directly onto the agar. As a result, the method retains key procedural elements that are more closely related to the classical spot test than to antibiotic agar diffusion assays. Nevertheless, our approach represents a deliberate workflow adaptation of the spot test to routine laboratory settings, enabling the use of established AST infrastructure and resources, providing a standardized and simplified workflow that can be readily adopted by routine diagnostic laboratories without requiring substantial changes to existing laboratory practice.

In contrast to small-molecule antibiotics, bacteriophages represent self-replicating biological particles whose behavior in agar is differently influenced by particle size, environmental conditions, media composition, and host–receptor interactions. These biological differences make phage testing inherently more susceptible to external factors and more prone to contamination effects than antibiotic testing.

Precisely for this reason, the aim of the Disk-PST is not to assess phage replication efficiency or diffusion properties, but to provide a robust, standardized, and purely attributive screening method for routine laboratories to determine whether lysis occurs or not under defined conditions. This includes its potential use as an initial screening tool for candidate phages as well as for verifying the activity of PTMPs following transport from a phage center to the testing laboratory.

To further facilitate standardized initial screening in routine laboratories and long-term storage of PTMPs, the use of ready-to-use phage-loaded materials would be even more advantageous. For liquid-based susceptibility testing, hydrogel- or polymer/sugar-derived solid ready-to-use formulations were proposed (38, 39). Similar approaches that are reviewed in the literature, based on polymers, nanoparticles, or fibers for different local application concepts are conceivable for the proposed agar-based method (40, 41). Furthermore, phage-antibiotic interactions could be investigated by combining PST and AST methods. Khong et al. proposed an agar-based technique to estimate additive, antagonistic, or synergistic phage-antibiotic interactions by preloading filter strips with phages or antibiotics, respectively, and measuring lytic zones (42).

In the future, a decentralized–centralized diagnostic model could be implemented. Initial Disk-PST screening with provided or commercially available PTMPs would be performed by routine laboratories, followed by the transfer of the clinical isolate and further testing to national phage reference centers. At these expert centers, more labor-intensive methods—such as kinetic assays, synergy testing with antibiotics, or survival assays—would be carried out. These steps would precede the production of a personalized PTMP or phage cocktail.

### Conclusion

This study represents the first inter-laboratory comparison of the previously described standardized agar-based Disk-PST, demonstrating high reproducibility, sensitivity, and agreement with the reference method (double agar overlay) across 12 routine laboratories in Germany. Our standardized Disk-PST offers an easily implementable and inexpensive tool for routine diagnostics. Although the interpretation of microcolonies and the phenomenon “lysis from without” remain potential sources of uncertainty, targeted training and validation assays can mitigate these effects. A decentralized–centralized diagnostic model could facilitate broad implementation in the future, with initial Disk-PST screening performed in routine laboratories and confirmatory testing performed in specialized reference centers. Harmonizing evaluation criteria will be crucial for diagnostic reliability and safe clinical application. Therefore, the Disk-PST is the first routine test for phage susceptibility determination that achieves excellent quality indicators.

## Data Availability

The original contributions presented in this study are included in the article and its supplemental material. Further inquiries can be directed to the corresponding author.

## SUPPLEMENTAL MATERIAL

Supplemental tables and figures jcm.00029-26-s0001.docx Tables S1 to S8 and Fig. S1 to S6.

Video S1 jcm.00029-26-s0002.mp4 Instructional video.

ASM does not own the copyrights to Supplemental Material that may be linked to, or accessed through, an article. The authors have granted ASM a non-exclusive, world-wide license to publish the Supplemental Material files. Please contact the corresponding author directly for reuse.
