# Supplementary material for: Toward routine implementation of phage testing: an inter-laboratory comparison of a disk-based susceptibility testing (Disk-PST)
Source: J Clin Microbiol. 2026 Oct 12;64(11):e00029-26. doi: 10.1128/jcm.00029-26 (PMC13753784; doi:10.1128/jcm.00029-26)
Supplement: Supplementary file 1 [file jcm.00029-26-s0001.docx]

**Supplemental Material**

Table S 1: Media, chemicals, and devices used in this study.

| **Type** | **Name** | **Description (supplier)** |
| --- | --- | --- |
| Chemicals | Agar | Agar technical no. 2 (Oxoid, Wesel, Germany) |
|  | CB | Caso Bouillon (Carl ROTH, Karlsruhe, Germany) |
|  | LB | Lennox LB broth base (Thermo Fisher Scientific, Waltham, MA, USA) |
|  | MgSO_4_ | Magnesium sulfate (Thermo Fisher Scientific, Waltham, MA, USA) |
|  | NaCl | Sodium chloride (Carl ROTH, Karlsruhe, Germany) |
|  | Tris-HCl | Tris hydrochloride (Carl ROTH, Karlsruhe, Germany) |
| Agar | CB bottom agar | 2.5 % Caso Bouillon, 1 % agar technical no. 2 (wt/vol), 5 mM MgSO_4_ |
|  | CB soft agar | 2.5 % Caso Bouillon, 0.4 % agar technical no. 2 (wt/vol) |
|  | Columbia agar | Columbia agar (BD Columbia agar with 5 % sheep blood; Becton Dickinson GmbH, Heidelberg, Germany) |
|  | LB bottom agar | 2.5 % Lennox LB broth base, 1 % agar technical no. 2 (wt/vol) |
|  | LB soft agar | 2.5 % Lennox LB broth base, 0.4 % agar technical no. 2 (wt/vol) |
|  | MH II agar | Mueller-Hinton II agar (BD Mueller-Hinton II agar, Becton Dickinson GmbH, Heidelberg, Germany) |
| Liquids | CB medium | 2.5 % Caso Bouillon |
|  | LB medium | 2.5 % Lennox LB broth base |
|  | Saline solution | 0.9 % NaCl (wt/vol) |
|  | SM buffer | 100 mM NaCl, 8 mM Mg SO_4_, 50 mM Tris-HCl (pH 7.5) |
| Devices | Culture swab | Culture swab (Σ-Transwab, Medical Wire & Equipment, Corsham, UK) |
|  | Disk | Whatman antibiotic disks, 6 mm (GE Healthcare, Chicago, IL, USA) |
|  | Drigalski spatulas | Inoculation spreader (SARSTEDT, Nuembrecht, Germany) |

Table S 2: Results of spot tests and phage susceptibility testing (PST) of clinical strains using Escherichia phage UMR_Zora (DSM 120226). Tests were performed in three biological replicates, each in technical duplicate (not shown). For subsequent analyses, samples with two or three positive biological replicates (2/3 or 3/3) were classified as positive, whereas samples with zero or one positive biological replicate (0/3 or 1/3) were classified as negative. * reference strain.

| **Strain** | ***Escherichia* phage UMR_Zora (DSM 120226)** | |
| --- | --- | --- |
|  | **Spot** | **PST** |
| **1** | 0/3 | 0/3 |
| **2** | 0/3 | 0/3 |
| **3** | 3/3 | 3/3 |
| **5** | 0/3 | 0/3 |
| **6** | 0/3 | 0/3 |
| **7** | 0/3 | 0/3 |
| **9** | 0/3 | 0/3 |
| **10** | 0/3 | 0/3 |
| **13** | 3/3 | 0/3 |
| **14** | 3/3 | 3/3 |
| **15** | 0/3 | 0/3 |
| **16** | 0/3 | 0/3 |
| **18** | 0/3 | 0/3 |
| **19** | 0/3 | 0/3 |
| **20** | 0/3 | 0/3 |
| **21** | 0/3 | 0/3 |
| **22** | 0/3 | 0/3 |
| **23** | 3/3 | 3/3 |
| **24** | 0/3 | 0/3 |
| **25** | 0/3 | 0/3 |
| **26** | 0/3 | 0/3 |
| **27** | 0/3 | 0/3 |
| **28** | 0/3 | 0/3 |
| **29** | 0/3 | 0/3 |
| **30** | 0/3 | 0/3 |
| **31** | 0/3 | 0/3 |
| **32** | 0/3 | 0/3 |
| **33** | 0/3 | 0/3 |
| **34** | 0/3 | 0/3 |
| **35** | 0/3 | 0/3 |
| **37** | 0/3 | 0/3 |
| **39** | 0/3 | 0/3 |
| **40** | 3/3 | 1/3 |
| **43** | 0/3 | 0/3 |
| **44*** | 3/3 | 3/3 |
| **45** | 0/3 | 0/3 |
| **46** | 0/3 | 0/3 |
| **49** | 0/3 | 0/3 |
| **50** | 0/3 | 0/3 |
| **51** | 0/3 | 0/3 |
| **52** | 0/3 | 0/3 |
| **53** | 0/3 | 0/3 |
| **54** | 0/3 | 0/3 |
| **55** | 0/3 | 0/3 |
| **56** | 0/3 | 0/3 |
| **57** | 0/3 | 0/3 |
| **58** | 0/3 | 0/3 |
| **59** | 0/3 | 0/3 |
| **60** | 3/3 | 3/3 |
| **61** | 0/3 | 0/3 |
| **65** | 0/3 | 0/3 |
| **66** | 0/3 | 0/3 |
| **67** | 0/3 | 0/3 |
| **71** | 0/3 | 0/3 |
| **72** | 0/3 | 0/3 |
| **73** | 0/3 | 0/3 |
| **74** | 0/3 | 0/3 |
| **75** | 0/3 | 0/3 |
| **76** | 0/3 | 0/3 |
| **77** | 0/3 | 0/3 |
| **78** | 0/3 | 0/3 |
| **79** | 0/3 | 0/3 |
| **80** | 0/3 | 0/3 |
| **81** | 0/3 | 0/3 |
| **82** | 0/3 | 0/3 |
| **83** | 0/3 | 0/3 |
| **84** | 0/3 | 0/3 |
| **85** | 3/3 | 3/3 |
| **86** | 0/3 | 0/3 |
| **87** | 0/3 | 0/3 |
| **88** | 0/3 | 0/3 |
| **89** | 0/3 | 0/3 |
| **90** | 0/3 | 0/3 |
| **91** | 3/3 | 3/3 |
| **92** | 0/3 | 0/3 |
| **93** | 0/3 | 0/3 |
| **94** | 0/3 | 0/3 |
| **95** | 0/3 | 0/3 |
| **96** | 0/3 | 0/3 |
| **97** | 0/3 | 0/3 |
| **98** | 0/3 | 0/3 |
| **99** | 0/3 | 0/3 |
| **100** | 0/3 | 0/3 |
| **101** | 0/3 | 0/3 |
| **102** | 0/3 | 0/3 |
| **106** | 0/3 | 0/3 |
| **107** | 0/3 | 0/3 |
| **108** | 3/3 | 3/3 |
| **109** | 0/3 | 0/3 |
| **110** | 0/3 | 0/3 |
| **111** | 0/3 | 0/3 |
| **112** | 0/3 | 1/3 |
| **113** | 0/3 | 0/3 |
| **114** | 0/3 | 0/3 |
| **115** | 0/3 | 0/3 |
| **116** | 0/3 | 0/3 |
| **117** | 0/3 | 0/3 |
| **118** | 0/3 | 0/3 |
| **119** | 0/3 | 0/3 |
| **120** | 0/3 | 0/3 |
| **121** | 0/3 | 0/3 |
| **122** | 3/3 | 3/3 |
| **123** | 0/3 | 0/3 |
| **124** | 0/3 | 0/3 |
| **125** | 0/3 | 0/3 |
| **126** | 0/3 | 0/3 |
| **127** | 0/3 | 0/3 |
| **128** | 0/3 | 0/3 |
| **129** | 0/3 | 0/3 |
| **130** | 0/3 | 0/3 |
| **131** | 0/3 | 0/3 |
| **132** | 3/3 | 3/3 |
| **133** | 0/3 | 0/3 |
| **134** | 0/3 | 0/3 |
| **135** | 0/3 | 0/3 |
| **136** | 0/3 | 0/3 |
| **137** | 0/3 | 0/3 |
| **138** | 0/3 | 0/3 |
| **139** | 0/3 | 0/3 |
| **140** | 0/3 | 0/3 |
| **141** | 0/3 | 0/3 |
| **142** | 3/3 | 3/3 |
| **143** | 0/3 | 0/3 |
| **144** | 3/3 | 3/3 |
| **145** | 0/3 | 0/3 |
| **146** | 0/3 | 0/3 |
| **147** | 0/3 | 0/3 |
| **148** | 3/3 | 3/3 |
| **149** | 0/3 | 0/3 |
| **150** | 3/3 | 3/3 |
| **151** | 0/3 | 0/3 |
| **DSM 5923** | 3/3 | 3/3 |
|  |  |  |
|  | No lysis zone in spot test/ PST | |
|  | Lysis zone in spot test | |
|  | Lysis zone in PST | |

Table S 3: Results of spot tests and phage susceptibility testing (PST) of strains using Staphylococcus phage UMR_Minerva (DSM 117679). Tests were performed in three biological replicates, each in technical duplicate (not shown). For subsequent analyses, samples with two or three positive biological replicates (2/3 or 3/3) were classified as positive, whereas samples with zero or one positive biological replicate (0/3 or 1/3) were classified as negative.* reference strain

| **Strain** | ***Staphylococcus* phage UMR_Minerva (DSM 117679)** | |  |
| --- | --- | --- | --- |
|  | **Spot** | **PST** |  |
| **2** | 3/3 | 3/3 |  |
| **3** | 3/3 | 3/3 |  |
| **4** | 0/3 | 0/3 |  |
| **5** | 3/3 | 3/3 |  |
| **6** | 0/3 | 0/3 |  |
| **7** | 3/3 | 0/3 |  |
| **8*** | 3/3 | 3/3 |  |
| **10** | 3/3 | 3/3 |  |
| **14** | 0/3 | 0/3 |  |
| **15** | 2/3 | 0/3 |  |
| **16** | 0/3 | 0/3 |  |
| **18** | 0/3 | 0/3 |  |
| **19** | 0/3 | 0/3 |  |
| **20** | 3/3 | 3/3 |  |
| **21** | 2/3 | 0/3 |  |
| **27** | 0/3 | 1/3 |  |
| **28** | 3/3 | 3/3 |  |
| **31** | 3/3 | 3/3 |  |
| **34** | 0/3 | 0/3 |  |
| **36** | 1/3 | 0/3 |  |
| **37** | 3/3 | 0/3 |  |
| **40** | 3/3 | 1/3 |  |
| **41** | 0/3 | 0/3 |  |
| **42** | 3/3 | 0/3 |  |
| **45** | 2/3 | 0/3 |  |
| **47** | 0/3 | 0/3 |  |
| **48** | 0/3 | 1/3 |  |
| **49** | 3/3 | 3/3 |  |
| **50** | 3/3 | 3/3 |  |
| **51** | 0/3 | 0/3 |  |
| **53** | 3/3 | 3/3 |  |
| **54** | 1/3 | 0/3 |  |
| **55** | 3/3 | 3/3 |  |
| **56** | 3/3 | 3/3 |  |
| **57** | 0/3 | 0/3 |  |
| **58** | 1/3 | 0/3 |  |
| **59** | 0/3 | 0/3 |  |
| **60** | 0/3 | 0/3 |  |
| **61** | 0/3 | 0/3 |  |
| **62** | 3/3 | 3/3 |  |
| **63** | 0/3 | 0/3 |  |
| **64** | 0/3 | 1/3 |  |
| **65** | 0/3 | 0/3 |  |
| **66** | 0/3 | 0/3 |  |
| **67** | 3/3 | 3/3 |  |
| **68** | 1/3 | 0/3 |  |
| **69** | 0/3 | 0/3 |  |
| **70** | 0/3 | 0/3 |  |
| **71** | 2/3 | 2/3 |  |
| **72** | 3/3 | 3/3 |  |
| **RP62A** | 3/3 | 3/3 |  |
|  |  |  |  |
|  | No lysis zone in spot test/ PST | | |
|  | Lysis zone in spot test | | |
|  | Lysis zone in PST | | |

Figure S 1: Stability of phage Minerva over 14 days in different buffers (SM buffer, SM buffer supplemented with glycerol [1:10], NaCl, and NaCl supplemented with glycerol [1:10]) at various storage temperatures (-80 °C, -20 °C, 4 °C, 21 – 24 °C). Phage titers were determined at 1, 3, 7 and 14 days using the double agar overlay assay and are presented as PFU/mL (mean ± SD).

Figure S 2: Stability of phage Zora over 14 days in different buffers (SM buffer, SM buffer supplemented with glycerol [1:10], NaCl, and NaCl supplemented with glycerol [1:10]) at various storage temperatures (-80 °C, -20 °C, 4 °C, 21 – 24 °C). Phage titers were determined at 1, 3, 7 and 14 days using the double agar overlay assay and are presented as PFU/mL (mean ± SD).

Table S 4: Comparison of error rates between biological replicates during In-house validation. (BR: Biological Replicate, CI: Confidence Interval)

| **BR** | **Total samples** | **Errors** | **Error rate** | **95 % CI** | ***z*-Score** | ***p*-Value** |
| --- | --- | --- | --- | --- | --- | --- |
| **1** | 336 | 4 | 1.19 % | 0.0 - 2.4 % | - | - |
| **2** | 336 | 24 | 7.14 % | 4.4 - 9.9 % | -3.86 | ***p* < 0.001** |

Table S 5: Comparison of error rates between technical replicates within each test day during In-house validation. (BR: Biological Replicate, CI: Confidence Interval, TR: Technical Replicate)

| **BR** | **TR** | **Total samples** | **Errors** | **Error rate** | **95 % CI** | ***z*-Score** | ***p*-Value** |
| --- | --- | --- | --- | --- | --- | --- | --- |
| **1** | **1** | 336 | 4 | 1.19 % | 0.0 - 2.4 % | - | - |
|  | **2** | 336 | 4 | 1.19 % | 0.0 - 2.4 % | 0.00 | *p* = 1.00 |
| **2** | **1** | 336 | 24 | 7.14 % | 4.4 - 9.9 % | - | - |
|  | **2** | 336 | 24 | 7.14 % | 4.4 - 9.9 % | 0.00 | *p* = 1.00 |

Table S 6: Deviation rates of the Disk-PST from the gold standard by test strain during In-house validation. Deviation rates of the Disk-PST from the gold standard by tested strain during In-house validation are displayed across two test days and replicates. Values are given as absolute numbers and percentages. Statistical significance was assessed using Fisher’s exact test. (BR: Biological Replicate, PST: Phage Susceptibility Test, TR: Technical Replicate)

| **Disk-PST** | | **Strain** | | | | **Total** | **Fisher’s exact** |
| --- | --- | --- | --- | --- | --- | --- | --- |
|  |  | **1** | **2** | **3** | **4** |  |  |
| **BR 1 / TR 1** | No Deviation | 84 (100.00 %) | 84 (100.00 %) | 84 (100.00 %) | 80 (95.24 %) | 332 (98.81 %) | ***p* = 0.015** |
|  | Deviation | 0  (0.00 %) | 0  (0.00 %) | 0  (0.00 %) | 4  (4.76 %) | 4  (1.19 %) |  |
| **BR 1 / TR 2** | No Deviation | 84 (100.00 %) | 84  (100.00 %) | 84  (100.00 %) | 80  (95.24 %) | 332  (98.81 %) | ***p* = 0.015** |
|  | Deviation | 0  (0.00 %) | 0  (0.00 %) | 0  (0.00 %) | 4  (4.76 %) | 4  (1.19 %) |  |
| **BR 2 / TR 1** | No Deviation | 82 (97.62 %) | 75  (89.29 %) | 74  (88.10 %) | 81  (96.43 %) | 312  (92.86 %) | ***p* = 0.029** |
|  | Deviation | 2  (2.38 %) | 9  (10.71 %) | 10  (11.90 %) | 3  (3.57 %) | 24  (7.14 %) |  |
| **BR 2 / TR 2** | No Deviation | 82 (97.62 %) | 75  (89.29 %) | 74  (88.10 %) | 81  (96.43 %) | 312  (92.86 %) | ***p* = 0.029** |
|  | Deviation | 2  (2.38 %) | 9  (10.71 %) | 10  (11.90 %) | 3  (3.57 %) | 24  (7.14 %) |  |

Table S 7: Deviation rates of the Disk-PST from the gold standard by phage during In-house validation. Deviation rates of the Disk-PST from the gold standard by phage during In-house validation are displayed across two test days and replicates. Values are given as absolute numbers and percentages. Statistical significance was assessed using Fisher’s exact test. (BR: Biological Replicate, PST: Phage Susceptibility Test, TR: Technical Replicate)

| **Disk-PST** | | **Phage** | | | **Total** | **Fisher’s exact** |
| --- | --- | --- | --- | --- | --- | --- |
|  |  | **A** | **B** | **C** |  |  |
| **BR 1 / TR 1** | No Deviation | 112  (100.00 %) | 109  (97.32 %) | 111  (99.11 %) | 332  (98.81 %) | *p* = 0.329 |
|  | Deviation | 0 (0.00 %) | 3 (2.68 %) | 1 (0.89 %) | 4 (1.19 %) |  |
| **BR 1 / TR 2** | No Deviation | 112  (100.00 %) | 109  (97.32 %) | 111  (99.11 %) | 332  (98.81 %) | *p* = 0.329 |
|  | Deviation | 0 (0.00 %) | 3 (2.68 %) | 1 (0.89 %) | 4 (1.19 %) |  |
| **BR 2 / TR 1** | No Deviation | 112  (100.00 %) | 90  (80.36 %) | 110  (98.21 %) | 312  (92.86 %) | ***p* = 0.001** |
|  | Deviation | 0 (0.00 %) | 22 (19.64 %) | 2 (1.79%) | 24 (7.14 %) |  |
| **BR 2 / TR 2** | No Deviation | 112  (100.00 %) | 90  (80.36 %) | 110  (98.21 %) | 312  (92.86 %) | ***p* = 0.001** |
|  | Deviation | 0 (0.00 %) | 22 (19.64 %) | 2 (1.79%) | 24 (7.14 %) |  |

Table S 8: Feedback results from inter-laboratory comparison, presented as absolute numbers with corresponding percentages in parentheses. Feedback forms were returned by each participating laboratory and answers were calculated as percentage of the whole participation group. Answers were evaluated as positive (yes), negative (no) or not applicable

| **Question** | **Yes** | **No** | **Not applicable** | **No Answer** |
| --- | --- | --- | --- | --- |
| **Were you familiar with methods for phage susceptibility testing beforehand?** | 15 (38.5 %) | 24 (61.5 %) |  |  |
| **Are you familiar with the methodology of disk-diffusion antibiotic susceptibility testing according to EUCAST?** | 38 (97.4 %) | 01 (2.6 %) |  |  |
| **Was the material delivered in impeccable condition?** | 39 (100 %) |  |  |  |
| **Were the materials and reagents clearly identifiable?** | 39 (100 %) |  |  |  |
| **Was the instructional video easy to understand?** | 24 (61.5 %) |  | 09 (23.1 %) | 06 (15.4 %) |
| **Was the test easy to perform according to the written standard operating procedure?** | 39 (100 %) |  |  |  |
| **Was the test outcome clearly interpretable?** | 36 (92.3 %) | 03 (7.7 %) |  |  |
| **Did you encounter any difficulties during the execution of the test? If yes, please specify.** | 06 (15.4 %) | 33 (84.6 %) |  |  |
| **Did you encounter any difficulties during the evaluation of the test and/or the completion of the evaluation form? If yes, please specify.** | 03 (7.7 %) | 33 (84.6 %) | 03 (7.7 %) |  |

Figure S 3: Flow-chart inter-laboratory comparison

Figure S 4: Standard Operating Procedure for Disk-Phage Susceptibility Testing

| **Name:** __________________  **Laboratory:** __________________  □ Technical Staff  □ Academic Staff  *(Please check the*  *appropriate box)*  **Legend:**    **+** : Lysis  - : No Lysis  ? : inconclusive   | **Please document the results in the figures with + (lysis), – (no lysis) or ? (inconclusive).**  **Experiment Day 1**  Date of performance: __________________ Date of evaluation: __________________    Strain 1 Strain 2 Strain 3 Strain 4  **Experiment Day 2**  Date of performance: __________________ Date of evaluation: ___________________    Strain 1 Strain 2 Strain 3 Strain 4 |
| --- | --- |

Figure S 5: Evaluation Sheet

| **Questions** | **Yes** | **No** | **Not applicable** |
| --- | --- | --- | --- |
| Were you familiar with methods for phage susceptibility testing beforehand? | □ | □ | □ |
| Are you familiar with the methodology of disk-diffusion antibiotic susceptibility testing according to EUCAST? | □ | □ | □ |
| Was the material delivered in impeccable condition? | □ | □ | □ |
| Were the materials and reagents clearly identifiable? | □ | □ | □ |
| Was the instructional video easy to understand? | □ | □ | □ |
| Was the test easy to perform according to the written standard operating procedure? | □ | □ | □ |
| Was the test outcome clearly interpretable? | □ | □ | □ |
| Did you encounter any difficulties during the execution of the test?  If yes, please specify. | □ | □ | □ |
| Did you encounter any difficulties during the evaluation of the test and/or the completion of the evaluation sheet?  If yes, please specify. | □ | □ | □ |

Figure S 6: Feedback Sheet
